# Impact of COVID-19 on tuberculosis notifications

**DOI:** 10.1590/S1678-9946202466037

**Published:** 2024-06-07

**Authors:** Talita Antunes Antoniolli Pontes, Fernando Fernandez-Llimos, Astrid Wiens

**Affiliations:** 1 Universidade Federal do Paraná, Programa de Pós-Graduação em Assistência Farmacêutica, Curitiba, Paraná, Brazil; 2 Universidade do Porto, Faculdade de Farmácia, Laboratório de Farmacologia, Porto, Portugal; 3 Universidade Federal do Paraná, Departamento de Farmácia, Curitiba, Paraná, Brazil

**Keywords:** Tuberculosis, COVID-19, Public health, Interrupted time series analysis

## Abstract

The COVID-19 pandemic has significantly impacted the control of diseases by overwhelming healthcare systems, and tuberculosis (TB) notifications may have been affected. This study aimed to analyze the impact of COVID-19 on TB notifications in the Sao Paulo State. This is a retrospective study examining TB notifications extracted from the TBweb database (Jan 2015 to Dec 2022). We conducted an interrupted time series (ITS) analysis of TB notifications using the declaration of the COVID-19 pandemic as the interrupting event (Bayesian causal impact analysis). A total of 177,103 notifications of TB incident cases were analyzed, revealing a significant decrease in 2020 (13%) and in 2021 (9%), which lost significance in 2022. However, changes were not associated with population density or the area of the regions. Future analyses of the effects of TB underdiagnosis might help describe the impact of underreporting on future TB incidence and mortality.

## INTRODUCTION

Tuberculosis (TB) is a mandatory notifiable disease that, despite effective treatment, continues to pose a significant global public health challenge^1,2^. In 2020, TB affected approximately 9.9 million people worldwide, resulting in 1.3 million deaths among individuals without HIV infection^3^. On a global scale, TB ranks second in mortality among infectious diseases, following COVID-19. From 2020 to 2021, COVID-19 caused 5.94 million deaths globally, with 704,897 reported in Brazil^4,5^.

In Brazil, the incidence of TB in 2019 was 37.9 cases per 100,000 inhabitants. However, during the first year of the COVID-19 pandemic in 2020, TB incidence decreased by 12.1%, reaching 33.3 cases per 100,000 inhabitants. Subsequently, TB incidence gradually increased to 34.9 and 36.3 cases per 100,000 inhabitants in 2021 and 2022, respectively^6^.

The reduction in TB incidence during the COVID-19 pandemic could be attributed to several factors. The pandemic had a profound impact on health services and systems, significantly affecting the diagnosis and management of various diseases. Healthcare professionals and facilities typically dedicated to TB care were temporarily redirected to serve COVID-19 patients^7^. These organizational changes, coupled with increased difficulties in the population's access to health services, may have led to a decline in the diagnosis of TB patients and an increase in treatment discontinuity^8^. The undiagnosed or inadequately treated TB patients in 2020 and 2021 are likely to impact TB transmission and mortality in the years to come^6^. This study aimed to assess the impact of COVID-19 on TB notifications in the Sao Paulo State, Brazil, via an interrupted time series analysis (ITS).

## MATERIALS AND METHODS

### Study design

An interrupted time series (ITS) analysis was conducted using data from notifications of patients diagnosed with TB in the Sao Paulo State from January 2015 to December 2022. A time series is a continuous sequence of observations collected at equally spaced time intervals. This series can be “interrupted” by the implementation of an intervention or an unscheduled event. In a traditional ITS, the secular trend of the time series before the interruption is compared with the trend after the interruption. Modern ITS studies compare the contrafactual time series, forecasted using the pre-interruption time series, with the actual post-interruption time series. These modern ITS designs are considered robust for inferring causality of the interruption in a specific time series^9,10^.

This study was conducted and reported following the Strengthening the Reporting of Observational studies in Epidemiology (STROBE) statement^11^. The research protocol received approval from the Research Ethics Committee of the Health Sciences Sector of the Federal University of Parana under approval Nº 5.817.023 (CAAE 64746122.6.0000.0102).

### Data collection

The administrative division of the Sao Paulo State was obtained from the Brazilian Institute of Geography and Statistics (IBGE). This division comprises 11 intermediate regions, which, in turn, consist of 53 immediate regions, further divided into 645 municipalities^12^. Population data at the municipality level and gross domestic product (GDP) were also retrieved from the IBGE database^13^.

To create a comparative time series expected to be unaffected by the analyzed interruption—the COVID-19 pandemic— birth rates were considered. Data on live births were obtained from the Brazilian Information System on Live Births (SINASC), developed by the Brazilian Ministry of Health^14^.

Incident cases of TB were obtained from the Epidemiological Surveillance Center "Prof. Alexandre Vranjac" (CVE), established by the State Health Department of Sao Paulo. The CVE established an online reporting system, TB-Web, for the control of tuberculosis patients. Anonymized data on TB incident cases from January 2015 to December 2022, recorded in the TB-Web, were provided by the CVE in a Microsoft Excel file. The data included the following information for each TB incident case: age at the time of diagnosis, sex at birth, skin color, municipality of residence, date of TB diagnosis, and outcome. The residence of incarcerated individuals was considered separately. All incident cases were included for analysis unless residence data was missing.

### Data analysis

The population data at the municipality level obtained from the IBGE were aggregated into immediate and intermediate region levels. The total GDP of each municipality was calculated by multiplying the GDP per capita by the population of the municipality. The total GDP for municipalities within each region was aggregated to obtain the overall GDP at immediate and intermediate region levels. Subsequently, the GDP per capita for each immediate and intermediate region was calculated by dividing the total GDP of the region by its population.

TB incident cases at the municipality level were aggregated at immediate region, intermediate region, and state levels. The incidence was calculated by dividing the incident cases reported in a specific territory for a given year by the population of that territory, expressed per 100,000 inhabitants. Associations between TB incidence and TB determinants (i.e., population density, GDP per capita) were analyzed at municipality level.

Monthly time series were established for each of these administrative units. The interruption was set in April 2020, resulting in a pre-interruption period from January 2015 to March 2020 (63 months) and a post-interruption series from April 2020 to December 2022 (33 months). The interrupted time series (ITS) analysis was conducted using R/RStudio with the CausalImpact package^15^. Based on the pre-interruption time series, CausalImpact package uses a Bayesian approach to infer the evolution of a counterfactual post-interruption time series. In the first plot (original), CausalImpact illustrates the actual time series (evolution of TB incident cases) as a solid line and the Bayesian estimate of the counterfactual series as a dashed line, with a band representing the 95% prediction interval. The second plot (pointwise) displays the point-by-point difference between the actual and counterfactual series, also with the 95% predictive interval. The third plot (cumulative) illustrates the cumulative difference between the two series. CausalImpact also provides estimates of the relative effect of the interruption (i.e., COVID-19 pandemic inception), presented as percentages with their respective 95% credible intervals.

The adjustment to a normal distribution was evaluated using the Kolmogorov-Smirnov (KS) test, supplemented by visual inspection of the quantile-quantile (QQ) plot. Descriptive analyses were conducted for continuous variables, with means and standard deviations (SD) or medians and interquartile ranges (IQR) presented based on their normality. Differences in continuous variables were assessed using non-parametric Mann-Whitney's test, and correlations between two continuous variables were examined using Spearman's rho statistics. The analyses were performed in SPSS v28, with significance set at p < 0.05.

## RESULTS

### Participants

A total of 177,103 cases of TB were reported and recorded in the TBWeb from 2015 to 2022. In 10 cases, the municipality was absent, resulting in 177,093 cases included for analysis. A total of 22,282 cases (12.6%) were reported from incarcerated individuals and could not be assigned to any municipality. The overall TB incidence in Sao Paulo State increased from 47.91 in 2015 to 53.68 per 100,000 in 2022, with a noticeable reduction in 2020 and 2021 (Table 1). Among the 645 municipalities in Sao Paulo State, 14 (2.2%)—with a total population of 97,649 residents—did not report any TB cases during the eight years of the study.

**Table 1:** Variability of tuberculosis incidence across immediate regions of Sao Paulo State.

Year	SP State*	SP State w/o imprisoned**				
		Overall	Median	IQR	Min.	Max.
2015	47.91	41.3	23.3	18.89 - 26.03	12.52	101.69
2016	47.56	40.51	23.24	18.58 - 29.01	8.22	106.79
2017	50.94	42.38	24.55	20.76 – 29.45	12.66	105.1
2018	51.1	44.18	24.93	18.85 - 29.9	8.16	114.75
2019	50.18	44.33	25	19.72 - 29.13	10.9	115.46
2020	44.49	39.41	21.32	18.05 - 26.6	8.8	103.63
2021	45.01	40.64	22.46	19.01 - 26.99	11.92	104.99
2022	53.68	48.88	26.63	23.68 - 32.03	14.27	129.51

* incidence of tuberculosis in the immediate regions of the Sao Paulo State;

** incidence of tuberculosis in the immediate regions of the Sao Paulo State, excluding data from Imprisoned people.

The mean age of reported TB cases was 38.9 years (SD = 16.3), with a higher prevalence among White people (40%; n = 69,978) and males (72%; n = 128,041). The most frequent outcomes among reported cases were as follows: 67.1% (n = 118,775) were treatment success, 13.1% (n = 23,208) abandoned treatment, and 8.5% (n = 13,296) resulted in death, with 3.5% (n = 6,280) attributed to deaths due to TB.

**Figure 1:**
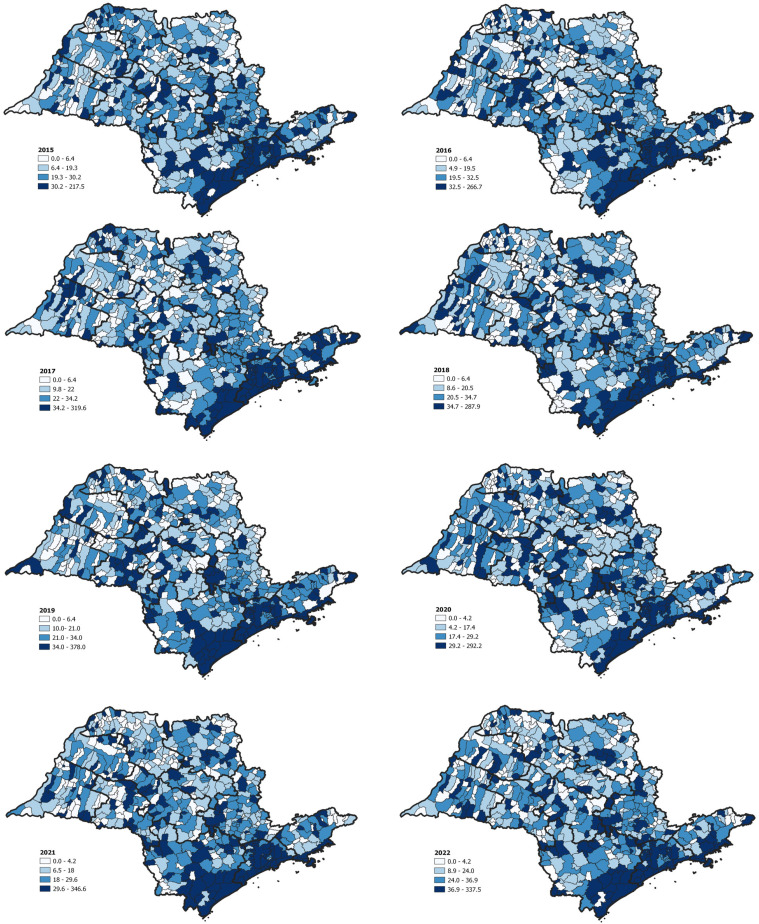
Incidence of tuberculosis in Sao Paulo State municipalities (excluding imprisoned population).

The incidence of TB at the municipality level (Figure 1) demonstrated a moderate-low association with population density but virtually no association with GDP (Table 2).

**Table 2:** Bivariate analysis of tuberculosis incidence determinants at municipality level.

Year	Population density		GDP per capita	
	rho*	p-value	rho*	p-value
2015	0.319	<0.001	0.096	0.016
2016	0.349	<0.001	0.107	0.007
2017	0.217	<0.001	-	0.796
2018	0.289	<0.001	-	0.141
2019	0.273	<0.001	0.102	0.010
2020	0.277	<0.001	0.117	0.003
2021	0.338	<0.001	-	.0152
2022	0.259	<0.001	-	0.354

* Spearman rho; GDP = gross domestic product.

**Figure 2:**
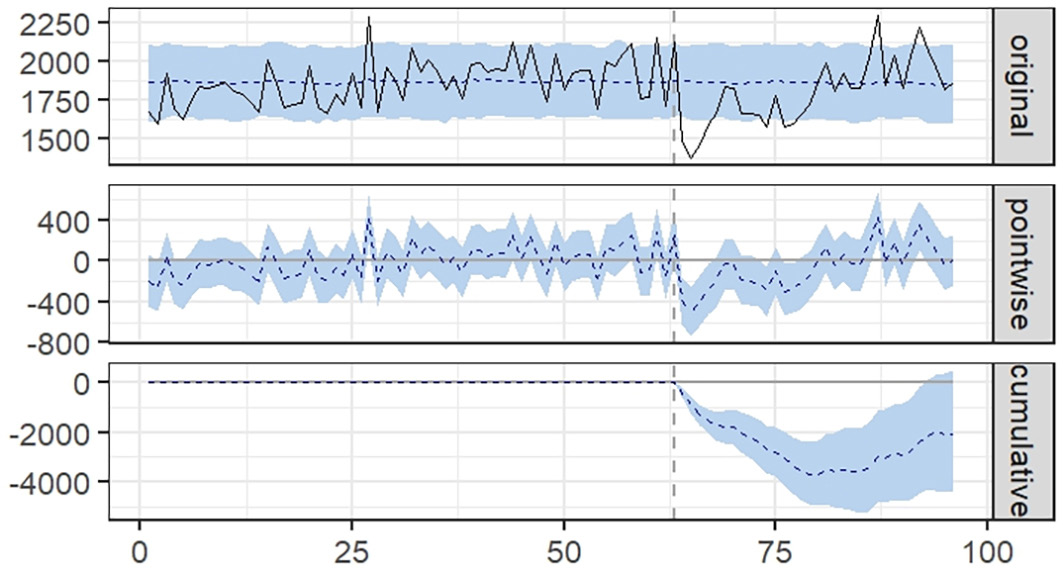
Interrupted time series analysis of tuberculosis notifications from 2015 to 2022.

The ITS analysis of TB notifications in Sao Paulo State from January 2015 to December 2022 revealed a clear impact of the COVID-19 pandemic on the overall incidence (Figure 2). There was a reduction of −13% (95%CI −17%, −9%) for 2020, −9% (95%CI −13%, −5%) for 2020–2021, which partially recovered for 2020–2022 to −3.3% (95%CI −7%, +0.85%).

**Figure 3:**
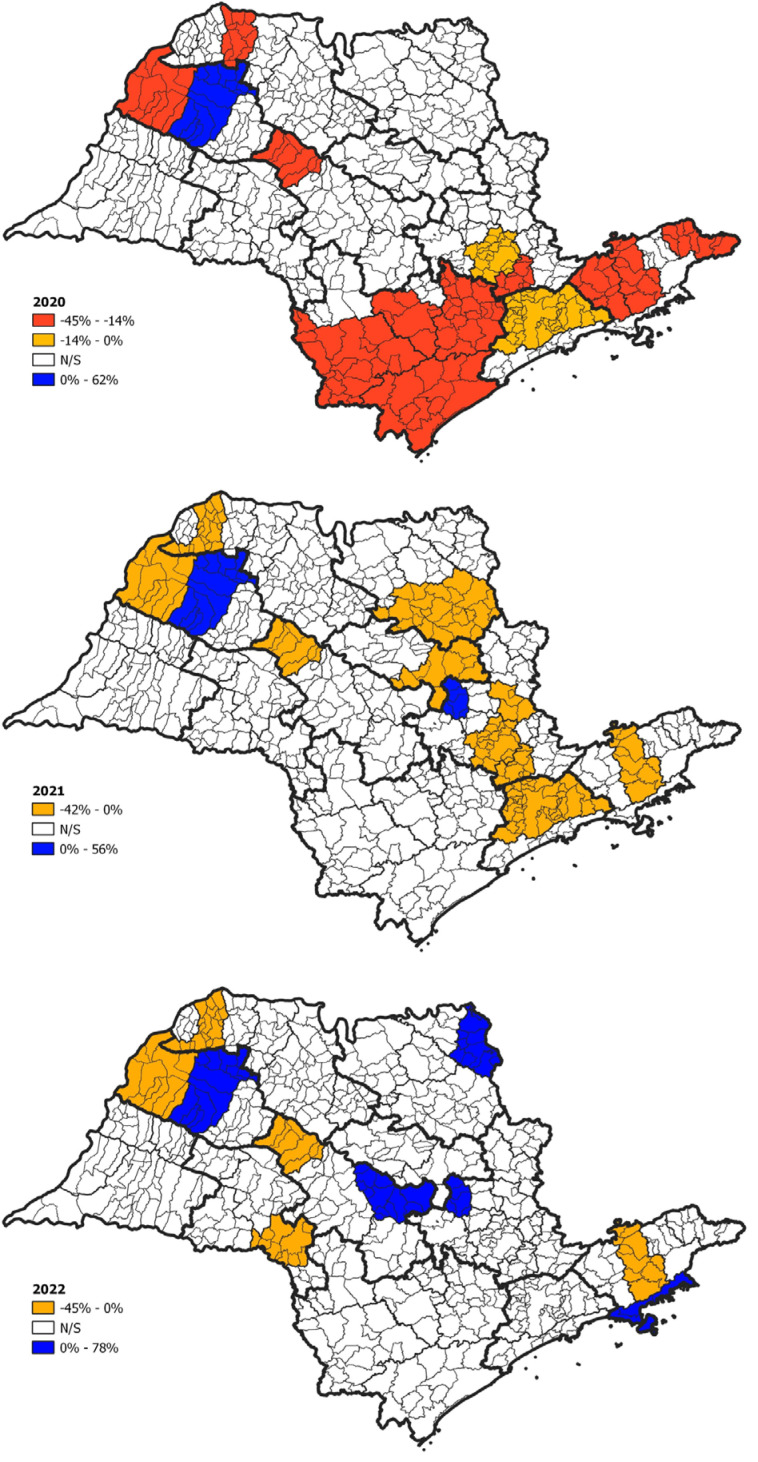
Impact of COVID-19 on tuberculosis incidence in Sao Paulo State at the immediate region level.

The impact of COVID-19 on TB incidence was not evenly distributed across the different immediate regions of Sao Paulo State (Figure 3). However, no association was found between the significant decrease in TB incidence reported for 2020 and surface area (Mann-Whitney, p = 0.090), GDP (p = 0.137), population (p = 0.179), or population density (p = 0.852).

## DISCUSSION

Our study evaluated TB incidence reported for Sao Paulo State from January 2015 to December 2022, and demonstrated the significant impact of COVID-19 on the notifications of this disease.

Cure and abandonment rates are major challenges for tuberculosis control. In this research, the treatment success rate reached only 67%, falling below the recommended threshold of 85%. Additionally, the abandonment rate exceeded 12%, which is 2.4 times higher than the maximum 5% considered acceptable. These suboptimal metrics pose a significant concern as they facilitate the emergence of resistant strains of *M. tuberculosis* ^16^, which is directly related to increased morbidity and mortality, and consequently the difficulty in controlling tuberculosis.

A wide range of variability in TB incidence was found across municipalities and regions (ranging from 8 to 100 cases per 100,000 residents). Regarding TB epidemiological analyses, Sao Paulo State is divided into four regions with different TB incidence^17^, ranging from 21.6 cases per 100,000 residents in inland regions to 91.5 in the Baixada Santista region in 2019. For studies using data from health databases, World Health Organization (WHO) recommends assessing data quality^18^. In our study, 14 municipalities comprising almost 100.000 residents in 2021 reported no TB case during the eight years studied. Considering the lowest regional TB incidence as a reference, these municipalities should have reported about 176 cases over these eight years (22 cases × 8 years). These considerations may raise concerns about the quality of the data in the TB surveillance and reporting system.

Despite mandatory TB reporting being considered a crucial milestone in the End TB Strategy, full implementation may not be sufficiently provided across the high-incidence countries^19^. TB underreporting has been demonstrated globally and Brazil is not an exception^20^. Underreporting of TB cases could be attributed to issues in three different phases of the case care line: access to the health system, TB diagnosis, and notification in the information system^21^. WHO considered that reduction of TB cases reported after COVID-19 is a mixture of underdiagnosis of people with TB and underreporting^1^. Before the COVID-19 pandemic, many countries had made significant progress in combating TB. However, these achievements were threatened by the pandemic scenario. According to WHO, more than 90% of countries experienced interruptions in the continuity of vital health services, leading to a significant reduction in the number of newly diagnosed and reported TB cases^22^. For the first time in more than a decade, mortality by TB increased in 2021, probably as a consequence of COVID-19 pandemics^1^. Hogan *et al*.^23^ estimated an increase of up to 20% in the number of deaths from TB in 2026 due to barriers in access to diagnosis and adequate care created during the pandemic period.

Negative impacts on disease data collection and reporting systems have been described during the COVID-19 pandemic. Data from more than 200 countries showed significant reductions in TB notifications, with drops of 25% to 30% reported in three high-burden countries (India, Indonesia, and the Philippines) from January to June 2020, compared to the same period in 2019^24^. Hino *et al*.^25^, in a scoping review, reported the challenges of social distancing in diagnosis, follow-up, and adherence to treatment, including the reorganization of tuberculosis services, mainly as a result of the necessary displacement of health teams to provide care for COVID-19. These reductions in case reporting could lead to a dramatic increase in TB-related deaths. These findings align with the results of our study. Other studies revealed a reduction in reported confirmed cases of pulmonary TB in all Brazilian regions except the North during the pandemic period. The Southeast (−8.2%), South (−8.9%), and Northeast (−10.9%) regions showed a percentage drop in notifications above the national average (−7.9%)^16,26^. Pontes *et al*.^27^ demonstrated a 36% drop in the number of TB notifications in a region of Sao Paulo State during 2020, with an emphasis on the months of July and August, when the peak of COVID-19 occurred in that region.

During the pandemic period, TB diagnosis, as well as reporting of newly diagnosed cases may have been hindered. According to Melo *et al*.^28^, the planning of epidemiological prevention and control actions is directly impacted by underreporting since data do not accurately reflect the epidemiological situation.

### Limitations

This study presents several limitations. The use of secondary data from health information systems is subject to bias and underreporting, which may be exacerbated by the pandemic situation. Municipal incidence rates were calculated using the resident population, which typically does not discount imprisoned residents. This might slightly underestimate the incidence at the municipality level. Additionally, notifications were derived from a single Brazilian state. As differences may exist with other regions, extrapolating the results is not recommended. Moreover, municipality surface area was calculated without exclusion of inhabited water sources and environmental preservation areas, which may hold a slight impact on different municipalities.

## CONCLUSION

Our study emphasizes the need to intensify TB prevention and control actions, starting with the reporting of diagnosed cases. The drop in reported TB incidence demonstrated in our interrupted time series (ITS) could be associated with lockdown and restrictive mobility measures. However, the rapid increase experienced during 2022 may reinforce the scenario of a reduction in diagnosis and reporting as a consequence of COVID-19-related challenges. Future studies should assess the impact of these findings on the incidence and mortality of TB.
